# Predicting 30-day mortality in patients with primary intracerebral hemorrhage: Evaluation of the value of intracerebral hemorrhage and modified new intracerebral hemorrhage scores

**Published:** 2018-01-05

**Authors:** Farzad Rahmani, Reza Rikhtegar, Alireza Ala, Aysan Farkhad-Rasooli, Haniyeh Ebrahimi-Bakhtavar

**Affiliations:** 1 Emergency Medicine Research Team, Tabriz University of Medical Sciences, Tabriz, Iran; 2 Neurosciences Research Center, Tabriz University of Medical Sciences, Tabriz, Iran; 3 Student Research Committee, Tabriz University of Medical Sciences, Tabriz, Iran

**Keywords:** Intracranial Hemorrhages, Mortality, Prognosis, Emergency Departments

## Abstract

**Background:** Different criteria have been proposed to determine the mortality rate of primary intracerebral hemorrhage (ICH). This study aimed to evaluate ICH and Modified New ICH scores in predicting 30-day mortality in patients with primary ICH.

**Methods:** In this prospective cohort study, 107 patients diagnosed with primary ICH were enrolled at an interval of six months (October 2015-March 2016). They were evaluated using Modified New ICH and ICH scores. The Modified New ICH score was different from the New ICH score since the National Institute of Health Stroke Scale (NIHSS) variables were replaced by Modified Rankin Scale (MRS) in the modified score.

**Results:** A total of 61 patients (57%) died, and 46 (43%) survived during the 30-day hospitalization. ICH ≥ 2 and Modified New ICH ≥ 3 scores predicted 30-day mortality rate in patients with the sensitivity and specificity rates of 87 and 63 percent, and 88 and 53 percent, respectively.

**Conclusion:** The current study showed that both ICH and Modified New ICH scores were almost equally effective in determining the mortality rate in patients with primary ICH, and both criteria had acceptable value in determining the mortality rate of patients. Therefore, routine assessment of ICH and Modified New ICH scores in patients with ICH in emergency wards is recommended.

## Introduction

Stroke is the fifth leading cause of death and long-term disability in the United States.^1^ Considering the poor prognosis, most of the families of patients with primary intracranial hemorrhage (ICH) often give their consent to the cessation of the treatment process.^2^^,^^3^ However, limiting treatment procedures for patients with ICH is associated with increased mortality rate in the short and long terms; therefore, physicians are recommended not to limit any of the treatment procedures in patients with ICH, because there may be a chance of recovery.^4^

Currently, there are some prognostic models for determining mortality rate and functional outcomes in patients with ICH. The ICH score is one of the simple and functional methods. This score is calculated by taking the patient's level of consciousness based on Glasgow Coma Scale (GCS), hemorrhage volume (cm^3^), presence or absence of intraventricular hemorrhage, hemorrhage site (supra or infratentorial), and patient's age (less or more than 80 years). Hemphill, et al.^5^ and Clarke, et al.^6^ concluded that the use of this score is convenient and practical, and could well predict the risk of mortality rate in the first minute of the patient’s visit.

Cheung and Zou^7^ introduced another scoring method for patients with non-traumatic ICH which is called New ICH score and based on the National Institute of Health Stroke Scale (NIHSS), initial body temperature, pulse pressure, and the presence of intraventricular hemorrhage and subarachnoid hemorrhage are determined.

Finally, considering the importance of the above discussion and determining the prognosis of patients with ICH as well as the lack of similar studies in our hospital, this study aimed to evaluate the performance of ICH and the Modified New ICH scores (new score) in predicting the 30-day mortality rate in patients with primary ICH.

## Materials and Methods

In the present prospective cohort study, a total of 107 patients with primary ICH who were referred and hospitalized in the emergency department of Imam Reza Hospital affiliated to Tabriz University of Medical Sciences, Tabriz, Iran, at an interval of six months (October 2015-March 2016) were enrolled. Sampling was done as a full census. Inclusion criteria included primary ICH (documented neural defects together with cerebral parenchyma bleeding with no evidence of trauma or history of surgery), and the consent of the patient or his/her relatives (in the case of disrupted consciousness). The exclusion criteria included cerebral hemorrhage caused by trauma, cerebral hemorrhage in brain tumors, hemorrhagic transformation of ischemic stroke, and aneurysm or vascular malformation. The current study was approved by the Ethics Committee of Tabriz University of Medical Sciences with ethical code of TBZMED.REC.1394.254 on 06.07.2015.

All patients who were diagnosed with signs and symptoms related to neurological problems such as loss of consciousness, paralysis or numbness of one side of the body, dizziness, and other cases were referred to the emergency department of Imam Reza Hospital and diagnosed with cerebral hemorrhage based on computed tomography (CT) scan images (Seimens, Somatom Emotion 6, Germany), were used for the study. The subjects received all standard support and treatment measures in accordance with the latest routine guidelines of the emergency and neurological departments.

Patients’ demographic information including gender, age, history of previous illnesses, and previous drug consumption were recorded. Then, criteria related to ICH (GCS, age, location of bleeding, presence of intraventricular hemorrhage, and bleeding volume) were recorded.^5^ Variables related to hemorrhage were recorded based on the brain CT scan results. Hemorrhage volume was calculated according to the ABC/2 formula. A, B and C, respectively indicated the largest diameter of the bleeding region in a stereotype slice in CT scan, in centimeter (cm), the diameter perpendicular to A in the same CT scan stereotypical image per cm, and the number of slices containing the bleeding multiplied by the distance between the slices in cm.

The variable used in the main New ICH score was NIHSS, which includes a total score of clinical examination of patients with sensory cases, lower motions, upper motions, facial paralysis, visual field, gaze, decreased level of consciousness, ataxia, dysarthria, and speech, and two-point discrimination.^7^ Considering that NIHSS score calculation is a complex process, the New ICH score was modified and simplified, and the variable, NIHSS was replaced with Modified Rankin Scale (MRS). MRS has 6 scores, ranging from 0 (asymptomatic patient) to 6 (deceased patient). MRS score is used to assess the severity of disability, and the dependency caused by stroke or other neurological disorders in performing everyday tasks. This criterion is frequently used in evaluating clinical outcomes of patients with stroke.^8^ In the present study, the MRS was calculated using the initial clinical assessment of patients. To calculate Modified New ICH score, variables including MRS, initial body temperature (using a mercury thermometer based on °C), pulse pressure (the difference between systolic and diastolic blood pressure), presence of ventricular hemorrhage, and presence of subarachnoid hemorrhage were obtained, and the related scores were calculated. NIHSS scoring was as follows: 0-10 (0 score), 11-20 (1 score), and 21-40 (2 score). To calculate the Modified New ICH score in this study, the MRS 0-2 score was used instead of NIHSS 0-10 score, NIHSS 11-20 equivalent to MRS 3-4 and NIHSS 21-40 equivalent to MRS 5. The cerebral hemorrhage location based on the brain anatomy and requiring surgery was recorded. After being hospitalized and receiving continued treatment, the patients were followed up in the hospital ward. The 30-day mortality was recorded.

All obtained data were entered into SPSS software (version 17, SPSS Inc., Chicago, IL, USA). Data description was done as mean ± standard deviation (SD), frequency, and percentage. Normal distribution of data was assessed using Kolmogorov-Smirnov test. Chi-square and independent t or U Mann-Whitney tests were used to respectively compare the qualitative and quantitative variables. Receiver operating characteristic (ROC) curve was used to determine the cut-off point, sensitivity, specificity, and predictive accuracy of mortality rate. Logistic regression was used to determine the prognostic value of variables in predicting mortality rate in patients. P value of less than 0.050 was considered significant in all the cases.

## Results

107 patients were enrolled the study. The mean age of patients was 63.83 ± 16.18 years. In terms of gender, there were 53 men (49.5%) and 54 women (50.5%). In terms of history of the disease, hypertension (69.0%, 74 patients), previous stroke (16.8%, 18 patients), and diabetes mellitus (14.0%, 15 patients) had the highest frequency among the patients, respectively. There were also other less prevalent diseases. Although, 69.0% of the patients had history of hypertension, only 51.4% (55 patients) of them were taking anti-hypertensive drug, and 9.3% (10 patients) of them were taking anticoagulant medication.

The number of deceased and survived patients were 61 (57%) and 46 (43%), respectively, within 30 days of hospitalization. The survival rate was 38.3% (41 patients) until discharge of patients. Table 1 shows comparison of the two groups of patients (with and without the 30-days mortality) in terms of the studied variables.

**Table 1:** Comparison of patients with and without 30-day mortality in terms of the studied variables

**Variables**	**With mortality**	**Without mortality**	**P**
Age (year) (mean ± SD)	70.75 ± 16.82	61.63 ± 13.82	0.003
Sex (Men/Women)	33/33	20/21	0.560
History of stroke (n) Positive Negative			> 0.999
	10	8	
	51	38	
Vital signs (mean ± SD)			
Heart rate (beat per minute) (mean ± SD)	84.62 ± 23.21	77.80 ± 13.40	0.780
Body temperature (°C) (mean ± SD)	36.97 ± 0.47	36.99 ± 0.36	0.800
Pulse pressure (mmHg) (mean ± SD)	76.46 ± 26.10	70.02 ± 19.57	0.160
GCS (mean ± SD)	7 ± 4	12 ± 3	˂ 0.001
ICH place (n) Supratentorial Infratentorial			0.786
	51	40	
	10	6	
IVH (n) Yes No			0.002
	40	16	
	21	30	
SAH (n) Yes No			0.109
	13	4	
	48	42	
Volume of bleeding (mean ± SD)	31.65 ± 18.93	18.67 ± 10.99	˂ 0.001
MRS (mean ± SD)	5 ± 1	3 ± 1	˂ 0.001
Lab findings (mean ± SD)			
Platelet (mm^3^) (mean ± SD)	242885.24 ± 92360.18	237369.56 ± 58628.91	0.724
PT (mean ± SD)	13.99 ± 2.77	14.09 ± 3.25	0.866
PTT (mean ± SD)	33.92 ± 13.03	31.46 ± 4.39	0.222
INR (mean ± SD)	1.23 ± 0.61	1.25 ± 0.64	0.814
Surgery need (n) Yes No			0.235
	16	7	
	45	39	
ICH score (mean ± SD)	2.90 ± 1.30	1.22 ± 0.81	˂ 0.001
Modified New ICH score (mean ± SD)	3.90 ± 0.98	2.56 ± 0.93	˂ 0.001

SD: Standard deviation; GCS: Glasgow Coma Scale; ICH: Intracerebral hemorrhage; IVH: Intraventricular hemorrhage; SAH: Subarachnoid hemorrhage; MRS: Modified Rankin Scale; PT: Prothrombin time; PTT: Partial thromboplastin time; INR: International normalized ratio

**Table 2:** Frequency of different anatomical sites of intracerebral hemorrhage in patients with or without the 30-day mortality

**Anatomic area of bleeding**	**With mortality (n)**	**Without mortality (n)**	**P**
Frontal lobe	7	1	0.005
Parietal lobe	0	4	
Temporal lobe	3	7	
Occipital lobe	0	2	
Thalamus	17	15	
Caudate	0	1	
Putamen	10	9	
Pons	6	2	
Cerebella	3	3	
Multiple place	15	2	

The patients were also compared in terms of the anatomic site of intracerebral hemorrhage. Table 2 shows the frequency of different anatomical sites of ICH in the two groups of patients (with and without the 30-day mortality).

The patients were evaluated in terms of ICH and Modified New ICH scores and, the mortality rate of patients was reported based on the obtained score. Table 3 shows the frequency of living and dead patients within 30 days of admission according to ICH and Modified New ICH scores.

**Table 3:** Frequency of living and dead patients within 30 days after admission according to intracerebral hemorrhage (ICH) and Modified New ICH scores

**Score**	**With ** **mortality ** **[n (%)]**	**Without ** **mortality ** **[n (%)]**
ICH score		
0	2 (18.2)	9 (81.8)
1	6 (23.1)	20 (76.9)
2	15 (50.0)	15 (50.0)
3	19 (90.5)	2 (9.5)
4	12 (100)	0 (0)
5	6 (100)	0 (0)
6	1 (100)	0 (0)
Modified New ICH score		
0	0 (0)	1 (100)
1	0 (0)	2 (100)
2	7 (25.0)	21 (75.0)
3	10 (40.0)	15 (60.0)
4	27 (81.8)	6 (18.2)
5	16 (94.1)	1 (5.9)
6	1 (100)	0 (0)

ICH: Intracerebral hemorrhage

As shown in table 3, all the patients died with an ICH score of 4 or higher. The mortality rate of score 0 was 18.2%. In the Modified New ICH score, the mortality rate of score 6 was 100%, and that of 0 and 1 was 0%. 

**Figure 1:**
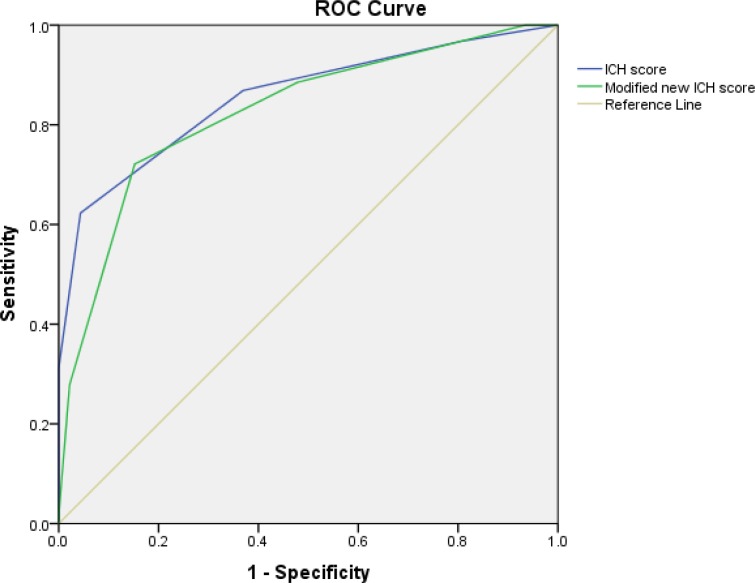
Receiver operating characteristic (ROC) curve of intracerebral hemorrhage (ICH) score and Modified New ICH score in predicting 30-day mortality in patients

ROC curve was used to evaluate the predictive value of the studied scores in determining the 30-day mortality rates in patients. The ROC curve (Figure 1) related to ICH and Modified New ICH scores in predicting 30-day mortality of patients. Based on the ROC curve, the value of the studied scores in determining the 30-day mortality in patients with primary ICH was calculated as shown in table 4.

**Table 4:** Predictive value of intracerebral hemorrhage (ICH) score and Modified New ICH score in predicting 30-day mortality in patients

**Variable**	**AUC**	**Cut-off ** **point**	**Sensitivity ** **(%)**	**Specificity ** **(%)**	**PPV**	**NPV**	**LR+**	**LR-**	**J** **point**
ICH score	0.855	≥ 2	87	63	0.70	0.83	2.35	0.21	0.50
Modified New ICH score	0.826	≥ 3	88	53	0.65	0.81	1.87	0.23	0.41

ICH: Intracerebral hemorrhage; AUC: Area under the curve; PPV: Positive predictive value; NPV: Negative predictive value; LR: Likelihood ratio

Logistic regression was used to determine the predictive value of age, volume of hemorrhage, and primary GCS and MRS in patients’ mortality. Patient’s age (P < 0.001), volume of hemorrhage (P = 0.048), and primary GCS (P = 0.001) were predictors of mortality in patients with ICH.

## Discussion

Scoring systems play an important role in determining the prognosis of different diseases during hospitalization.^9^^-^^11^ Determining the prognosis and prediction of the mortality rate in patients with ICH on arrival at the emergency ward plays an important role in the medical staff’s decision-making, and determining the most appropriate treatment program for patients and their families.^12^^,^^13^

The results obtained in the current study showed that factors such as patient's age, the initial level of consciousness by initial GCS, and the hemorrhage volume were factors that predicted the mortality of patients.

In a study, Lisk, et al.^14^ concluded that the most important predictors of prognosis in these patients were bleeding volume, age, ventricular expansion, and patient’s GCS. In another study, Juvela^15^ reported factors including low GCS, hypertension, smoking, intraventricular hemorrhage, surgery, cerebral hematoma, age, and the amount of alcohol consumed in a week prior to hemorrhage as the most important predictors. Fric-Shamji, et al.^16^ found no relationship between the volume of primary ICH and mortality, while other studies such as the present one showed a relationship between the volume of primary ICH and the spread of hemorrhage to cerebral ventricles with outcome in patients with ICH; mortality rate was related to volume of hemorrhage or ventricular hemorrhage as well.^17^^-^^19^

Hemphill, et al.^5^ concluded that the use of this score is convenient and practical, and could predict the risk of mortality rate from the moment the patient arrives the hospital very well. In their study, like the current one, all the patients with an ICH score of 4 or higher, died.

The primary ICH score has been introduced as an accurate factor predicting the mortality rate in patients with ICH.^5^^,^^20^^,^^21^ In another study, Hemphill, et al.^22^ also showed that the ICH score possesses high accuracy in predicting the long-term survival of patients (12 months). In a study that investigated the score, Cho, et al.^23^ showed that the cut-off point of New Modified ICH score was ≥ 3, with sensitivity and specificity of 76.3 and 98.8 percent, respectively, to help clinician decide on either to follow conservative treatment or proceed to surgery.

Fernandes, et al.^24^ concluded that all the patients with an ICH score of 5 or 6 died, and also reported the adverse outcomes rate of 100% in patients with ICH score above 5. 

The present study also evaluated the value of ICH and Modified New ICH scores in determining the 30-day mortality rate of patients with ICH. The Modified New ICH score was different from the New ICH score; since in the modified score, the MRS variable was used instead of NIHSS. The ICH score of ≤ 2 and the Modified New ICH score of ≤ 3 predicted the 30-day mortality rate of patients with sensitivity and specificity of 63% and 87%, and 88% and 53%, respectively. Although, the New ICH score was modified in the present study, acceptable results on predicting the outcome were obtained at the end. This score was modified in order to simplify its use. MRS is calculated more easily when compared with NIHSS. Besides, scores of 0 and 6 of the MRS were deleted in the initial calculation. 

One of the limitations of the present study was the lack of long-term follow-up of patients after discharge from the hospital. In addition, the NIHSS variable was not calculated in the patients.

## Conclusion

The present study showed that both ICH and Modified New ICH scores were almost equally effective in determining the mortality rate in patients with ICH, and both criteria had acceptable value in determining the mortality rate. Considering some different findings and lack of definitive results reported in previous studies, it is recommended to conduct similar studies as well as review studies in the field by taking into account other factors such as underlying diseases, patient’s selection procedure, etc. in order to obtain better results. It is also recommended that similar studies on the use of these criteria in other health centers be carried out, as well as evaluation of ICH and Modified New ICH scores as one of the measures used routinely in patients with ICH in emergency wards. Besides, considering that the new ICH score was modified for the first time in order to make it easier and practical, and the NIHSS variable was replaced by primary MRS, and similar results were obtained, it is recommended that this new score be evaluated in future studies.

## Notes:

**How to cite this article:** Rahmani F, Rikhtegar R, Ala A, Farkhad-Rasooli A, Ebrahimi-Bakhtavar H. Predicting 30-day mortality in patients with primary intracerebral hemorrhage: Evaluation of the value of intracerebral hemorrhage and modified new intracerebral hemorrhage scores. Iran J Neurol 2018; 17(1): 47-52.
